# Bibliometric analysis of research trends in stem cell therapy for knee osteoarthritis over the period 2001–2021

**DOI:** 10.3389/fcell.2022.996273

**Published:** 2022-10-18

**Authors:** Runzhi Chen, Yanyan Jiang, Laiya Lu, Pei Wang, Dongya Huang, Jingyi Wang, Zheng Liu, Shaojie Qin, Feng Yin

**Affiliations:** ^1^ School of Medicine, Tongji University, Shanghai, China; ^2^ Department of Joint Surgery, Shanghai East Hospital, School of Medicine, Tongji University, Shanghai, China; ^3^ Department of Critical Care Medicine, Renji Hospital, School of Medicine, Shanghai Jiao Tong University, Shanghai, China; ^4^ Department of Neurology, Shanghai East Hospital, School of Medicine, Tongji University, Shanghai, China

**Keywords:** bibliometric analysis, hotspot, knee osteoarthritis, stem cells, VOSviewer

## Abstract

Stem cell therapy is a promising treatment for knee osteoarthritis, but few bibliometric studies have been performed on the subject. Bibliometric analysis is helpful for identifying the most influential studies in a specific field and can evaluate the global research trends in stem cell therapy for knee osteoarthritis. The Web of Science Core Collection was searched for publications from 2001 to 2021. Publication performance was analyzed using several bibliometric parameters, including VOSviewer, to identify the research landscape of trends in topics, and CiteSpace was investigated to identify the keywords that have the strongest citation bursts. From 2001 to 2021, in total, 1,345 publications explored the research on stem cells in knee osteoarthritis. The United States contributed the largest number of publications and at the top list of international collaborations. Tokyo Medical and Dental University ranked first among institutions in the overall number of articles and citations. The journal of *Osteoarthritis and Cartilage* had the largest number of publications. Sekiya Ichiro was the most cited author, with 32 articles. The keywords with the most frequent occurrence were “osteoarthritis,” “mesenchymal stem cells,” and “cartilage,” in descending order of frequency. “fibroblast growth factor” and “extracellular vesicle” were the first and last searched theme terms, respectively. The number of publications on stem cells for knee osteoarthritis stays growing. Cartilage repair and paracrine function are current research hotspots for the stem cell therapy mechanism. Stem cell therapy has gradually advanced from basic research to the clinical application stage.

## Introduction

Osteoarthritis (OA), the most common type of arthritis among adults, is a leading cause of joint pain, body function loss, and disability. It is the fourth leading cause of disability and an important factor affecting disability-adjusted life quality and duration worldwide (Brooks, 2002; Fransen et al., 2011). Due to advances in research, knee osteoarthritis (KOA) is no longer conceived as a simple degeneration of cartilage. Instead, it involves subchondral bone changes, superfluous bone formation, synovial inflammation, and ligament and muscle changes as well (Kolasinski et al., 2020). Stem cells can migrate through the vascular endothelium or complete the mesenchymal migration to reach specific sites in the body, which may be part of the process of repair for cartilage damage (Laird et al., 2008). The therapeutic action of stem cells includes properties such as a unique self-renewal capacity that enables limitless proliferation and differentiation (Carstairs and Genever, 2014). Mesenchymal stem cells (MSCs), also known as mesenchymal stromal cells, are gaining popularity as a disease-modifying treatment due to their easy-harvesting feature, high safety, and potential to differentiate into cartilage tissue (Vezina et al., 2013). Furthermore, MSCs release several growth factors and cytokines to generate paracrine, anti-inflammatory, and immunomodulatory effects (Vega et al., 2015). Since the pathophysiology of OA is based on both degeneration and inflammation, the paracrine effect, reduction of the immune response, and stimulation of local tissue repair with the help of properties of MSCs, forming a disease-modifying treatment as a whole, would be conducive to the improvement of an intra-articular environment (Wei et al., 2014; Bastos et al., 2020). CiteSpace is a unique and influential web-based Java application used for data analysis and visualization (Chen, 2004). This software includes co-citations, co-authors, and co-occurrence keywords to provide suggestions for analyzing a certain research area (Chen and Leydesdorff, 2014). VOSviewer takes a holistic view of all research objects and explores research topics in an entire field by selecting different opinions (van Eck et al., 2010). However, few systematic reviews and studies of the literature on stem cell therapy have been conducted for KOA, especially in terms of quantitative and visual analysis of the literature. Using the Web of Science Core Database, the procedures of visualization analysis on the knowledge map in this article are as follows: first, Citespace was used to analyze the basic overview of the research on stem cell therapy for KOA. Then, hotspots, themes, and cutting-edge trends across the entire research field were explored through a co-occurrence map combining the burst therm terms function and VOS-viewer clustering function.

## Materials and methods

### Search strategy

Search terms were chosen from the list of Medical Subject Headings (MeSH) of the Institutes of Health (Table 1). All data about publications on stem cell therapy for KOA were obtained from Web of Science Core Collection by using the following search strategy to identify publications from 2001 to 2021. The search term TS = (stem cells OR cell, stem OR cells, stem OR stem cell OR progenitor cells OR cell, progenitor OR cells, progenitor OR progenitor cell OR mother cells OR cell, mother OR cells, mother OR mother cell OR colony-forming unit OR colony forming unit OR colony-forming units OR colony forming units) AND TS = (osteoarthritis, knee OR knee osteoarthritis OR koa OR osteoarthritis of knee OR osteoarthritis of the knee) were entered in the search field, with the language being refined to English only in the literature search and the inclusion criteria being “articles.” All searches were conducted on 21 November 2021 to avoid potential bias caused by database updates. Four experienced investigators in the field of joint surgery extracted all relevant data, including publication, author, title, abstract, keywords, source, language, citation, etc. They downloaded the abovementioned data in a text format from the WoS Core Collection and imported them into Citespace, VOS viewer, and a bibliometric tool for further analysis.

**TABLE 1:** Search terms divided into two categories, stem cells and osteoarthritis, knee.

Category	Search terms
Stem cells	“cell, stem” “cells, stem” “stem cell” “progenitor cells” “cell, progenitor” “cells, progenitor” “progenitor cell” “mother cells” “cell, mother” “cells, mother” “mother cell” “colony-forming unit” “colony forming unit” “colony-forming units” “colony forming units”
Osteoarthritis, knee	“knee osteoarthritis” “knee osteoarthritis” “osteoarthritis of knee” “osteoarthritis of the knee”

### Bibliometric analysis

WoS Core Database and Microsoft Excel (Microsoft 365 version) were used to describe the characteristics of the publications, including the total and annual number of publications; publication sources, to illustrate, whether they are at national, institutional, or individual levels, as well as the research field distributions. Overlay visualizations of networks connecting authors, organizations, and countries as well as keyword occurrence were generated using VOS viewer version 1.6.16 software (Leiden University, Leiden, Netherlands). Co-cited references from clustering analysis, a timeline view, and analysis of strongest citation bursts and keywords’ strongest citation bursts were conducted to show the time scale of themes by CiteSpace 5.7R5 (Chen Meichao, Drexel University). The visual knowledge network created by CiteSpace and VOS Viewer consists of nodes and lines. The numbers of publications, citations, and keyword occurrences are represented by circles of various sizes, and the thickness of the connecting lines represents the link strength. The start time of the research topic is represented by the color temperature in the network graph. In addition, the network is created by CiteSpace, in which each node is represented by a series of citation rings representing different years and the thickness of such rings is proportionate to the citation count in the corresponding time zone. The purple ring represents centrality, which identifies and measures the importance of the node. Nodes with high centrality are considered the key points connecting the preceding and the following parts of the network (Liang et al., 2017).

## Results

### Bibliometric analysis of publication output

#### Growth trend of publications

**FIGURE 1:**
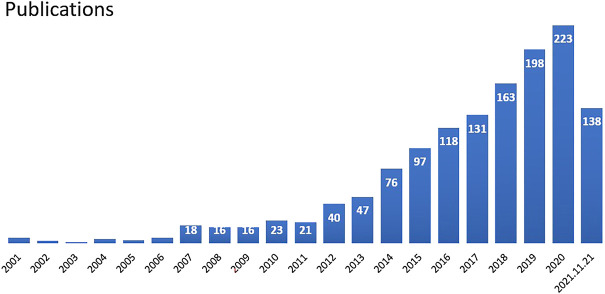
Annual outputs of publications regarding stem cell therapy for KOA and growth trends of global.

**FIGURE 2:**
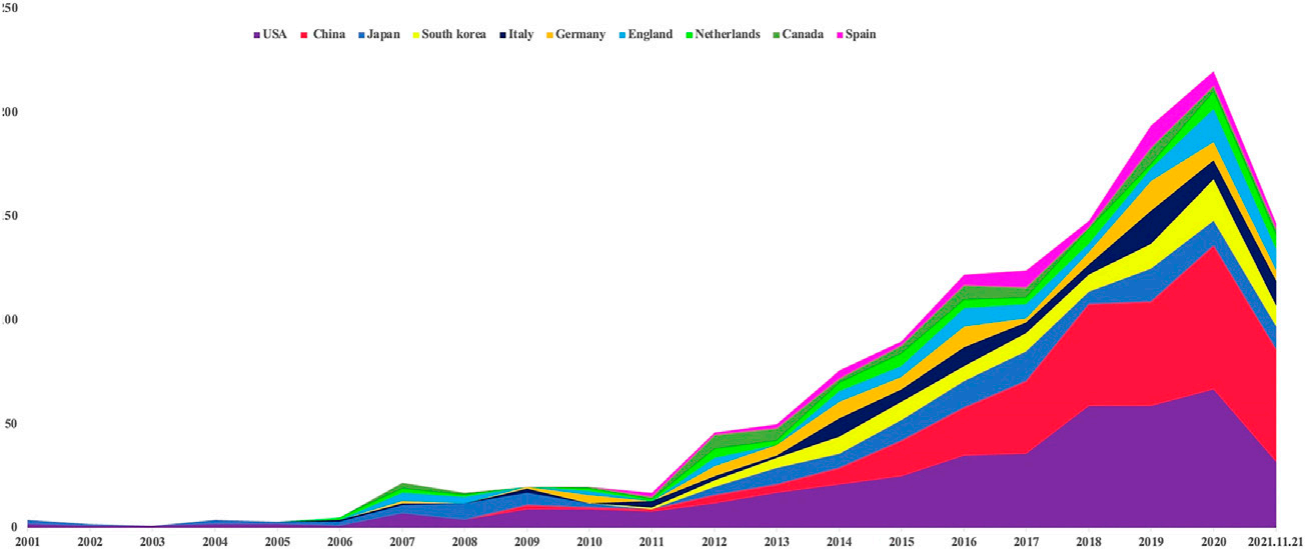
Top 10 most productive countries. Countries are indicated by colors, where the width of the color plate indicates annual publications per country.

Annual variation in the literature indicates trends in research development in this field, with the quantity serving as an important indicator of hotspot evaluation. The search strategy in this study helped identify 1,345 publications on stem cell research of KOA from the WoSCC. If one publication was co-authored by investigators from more than one country/region or institution, it was assigned equally to all participating parties. The yearly output between 2001 and 2021 is shown in Figure 1. The growth rate increased for some consecutive years, especially following 2014. In the WoSCC database, it can be seen that 69 countries or regions contributed to publications on stem cell research of KOA. The top 10 contributing countries are presented in Figure 2, among which the United States (United States) is the most prolific publisher.

#### Contribution of institutions and journals

Among 1857 organizations which have published relevant articles, and 142 produced over 5 publications each. Tokyo Medical and Dental University (Tokyo Med & Dent Univ) boasted the largest number of publications and citations (36 publications, cited 1,426 times). Zhejiang University (Zhejiang Univ) (27 publications, cited 787 times) and Hospital for Special Surgery (Hosp Special Surg) (37 publications, cited 872 times) ranked second and third, respectively. Of the 4,639 journals that published relevant articles, 420 produced over 20 publications each. *Osteoarthritis and Cartilage* had the most publications (62 publications, cited frequency 3861), followed by *American Journal of Sports Medicine* (47 publications, cited 1,486 times) and *Journal of Orthopaedic Research* (37 publications, cited 872 times). Universities and affiliated hospitals were the main institutions for the publication of stem cell research on KOA, with universities accounting for the largest proportion.

#### Contribution of authors

Sekiya Ichiro at the Section of Cartilage Regeneration, Graduate School, Tokyo Medical and Dental University, published 32 articles and was the most frequently cited author (cited 1,008 times). In second place was Hideyuki Koga (26 articles, cited 673 times) at the Oslo Sports Trauma Research Center, Norwegian School of Sports Sciences; next came Takeshi Muneta (21 articles, cited 888 times) at the Department of Orthopaedic Surgery, Tokyo Medical and Dental University, and Yong Gon Koh (19 articles, cited 1,211 times) at the Joint Reconstruction Center, Department of Orthopedic Surgery. Last of these were Yonsei Sarang Hospital and Kunikazu Tsuji (19 articles, cited 454 times) at the Department of Cartilage Regeneration, Tokyo Medical and Dental University. The ranking of the top 10 institutions (≥5 articles), journals, article authors, and reference citations is presented in Table 2.

**TABLE 2:** Top 10 institutions, journals, authors of publications, and reference articles on stem cell therapy for KOA.

Rank	Institution	Publications	Citations	Journal	Publications	Citations	Author	Publications	Citations	Cited references	Citations
1	Tokyo Med & Dent Univ	36	1,426	*Osteoarthritis and Cartilage*	62	3861	Ichiro Sekiya	32	1,008	CH Jo, 2014, Stem Cells	179
2	Zhejiang Univ	27	787	*American Journal of Sports Medicine*	47	1,486	Hideyuki Koga	26	673	M Brittberg, 1994, New Engl J Med	155
3	Hosp Special Surg	23	387	*Journal of Orthopaedic Research*	37	872	Takeshi Muneta	21	888	M Dominici, 2006, Cytotherapy	153
4	Yonsei Sarang Hosp	21	1,282	*Cartilage*	35	379	Yong Gon Koh	19	1,211	MF Pittenger, 1999, Science	144
5	Shanghai Jiao Tong Univ	20	803	*Scientific Reports*	32	539	Kunikazu Tsuji	19	454	JM Murphy, 2003, Arthritis Rheum-US	138
6	Rush Univ	19	202	*Plos One*	28	624	Mitsuru Mizuno	16	175	KKPH Pritzker, 2006, Osteoarthr Cartilage	105
7	Mayo Clin	17	870	*Stem Cell Research & Therapy*	27	645	Nobutake Ozeki	14	137	Y Sakaguchi, 2005, Arthritis Rheum-US	105
8	Peking Univ	17	279	*Arthritis Research & Therapy*	22	816	Jorge Chahla	12	247	A Vega, 2015, Transplantation	101
9	Cleveland Clin	16	205	*International Journal of Molecular Sciences*	22	365	Hisako Katano	11	81	S Wakitani, 2002, Osteoarthr Cartilage	91
10	Univ Pittsburgh	16	609	*Stem Cells Translational Medicine*	22	1,057	Yong Sang Kim	11	376	YG Koh, 2013, Arthroscopy	86

### Bibliometric analysis of co-authorship

#### Co-authorship of countries

**FIGURE 3:**
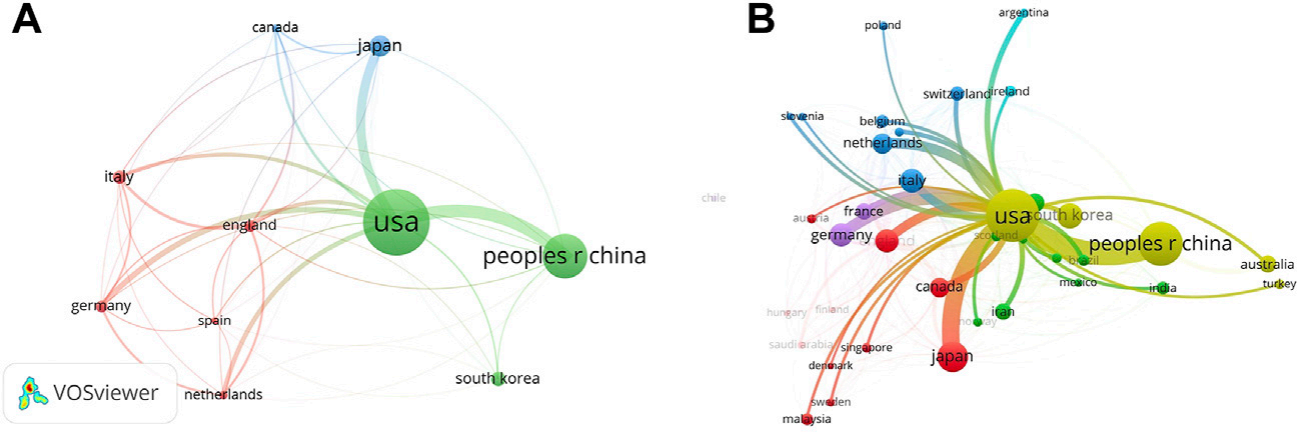
Co-authorship analysis of countries/regions regarding stem cell therapy for KOA research. **(A)** Relationships among the top 10 countries. **(B)** Co-authorship for the United States.

The relationships between the top 10 countries by the number of co-authored publications are shown in Figure 3A. The United States conducted the most active cooperation with other countries (33 links; total link strength: 232), followed by the UK (30 links; total link strength: 100), China (22 links; total link strength: 88), Germany (32 links; total link strength: 86), Italy (28 links; total link strength: 85). The main partners of the United States were China (link strength: 43), Japan (link strength: 31), Germany (link strength: 19), the Netherlands (link strength: 16), and the UK (link strength: 14) (Figure 3B).

#### Co-authorship of institutions

**FIGURE 4:**
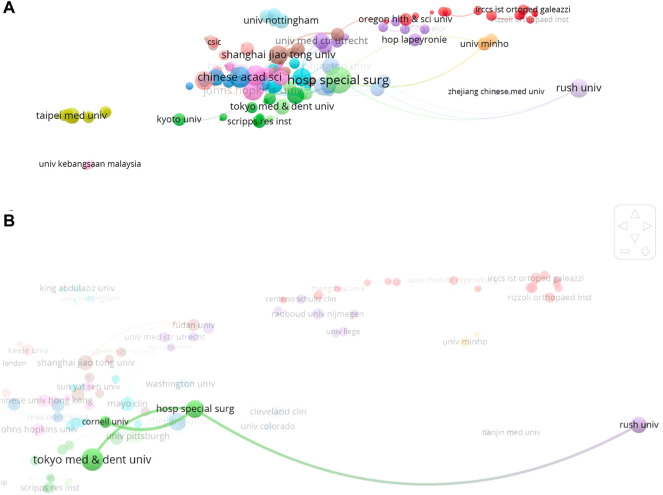
**(A)** Co-authorship analysis of institutions regarding stem cell therapy for KOA research. **(B)** Co-authorship for Hosp Special Surg.

The relationships of institutions are shown in Figure 4A. Hosp Special Surg showed the most active cooperation (22 links; total link strength: 36), followed by the Chinese Academy of Sciences (Chinese Acad Sci) (13 links; total link strength: 24), Chinese University of Hong Kong (Chinese Univ Hong Kong) (15 links; total link strength: 23), and Johns Hopkins University (Johns Hopkins Univ (14 links; total link strength: 23) by descending order. The main partners of Hosp Special Surg were Cornell University (Cornell Univ) (link strength: 5), Rush University (Rush Univ) (link strength: 3), and Tokyo Med & Dent Univ (link strength: 3) (Figure 4B).

#### Co-authorship of authors

**FIGURE 5:**
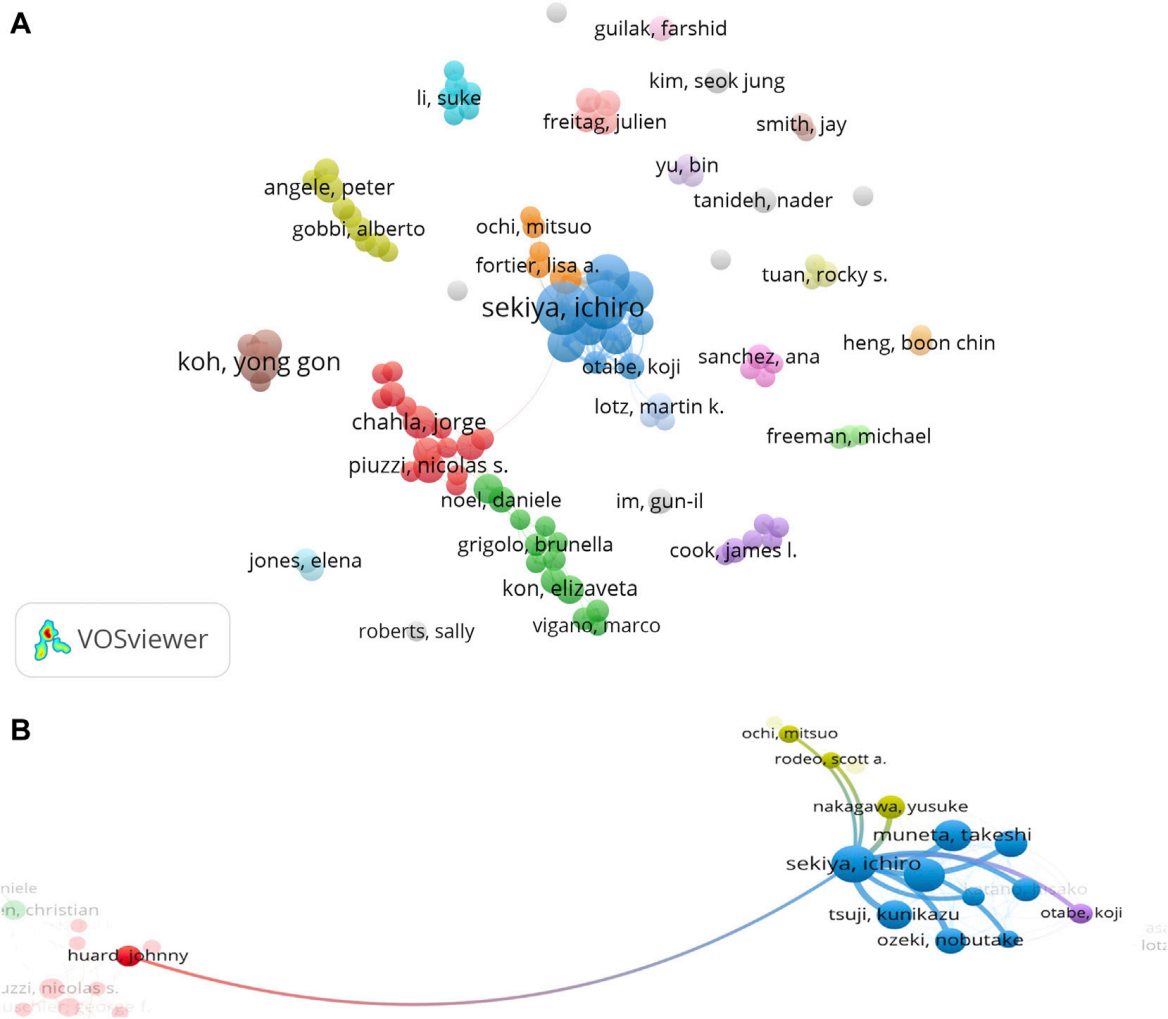
**(A)** Co-authorship analysis of authors regarding stem cell therapy for KOA research. **(B)** Co-authorship of Ichiro Sekiya.

The interaction between authors is shown in Figure 5A). Ichiro Sekiya showed the most active cooperation (13 links; total link strength: 139), followed by Hideyuki Koga (10 links; total link strength 127), Takeshi Muneta (10 links; total link strength: 94), and Kunikazu Tsuji (10 links; total link strength: 93). The main partner of Ichiro Sekiya was Hideyuki Koga (link strength: 26), Takeshi Muneta (link strength: 21), Tsuji, Kunikazu (link strength: 19), Mitsuru Mizuno (link strength: 15), and Nobutake Ozeki (link strength: 14) (Figure 5B).

## Bibliometric analysis of keywords co-occurrence, theme term trends, and keyword burst value

### Bibliometric analysis of keyword co-occurrence

**FIGURE 6:**
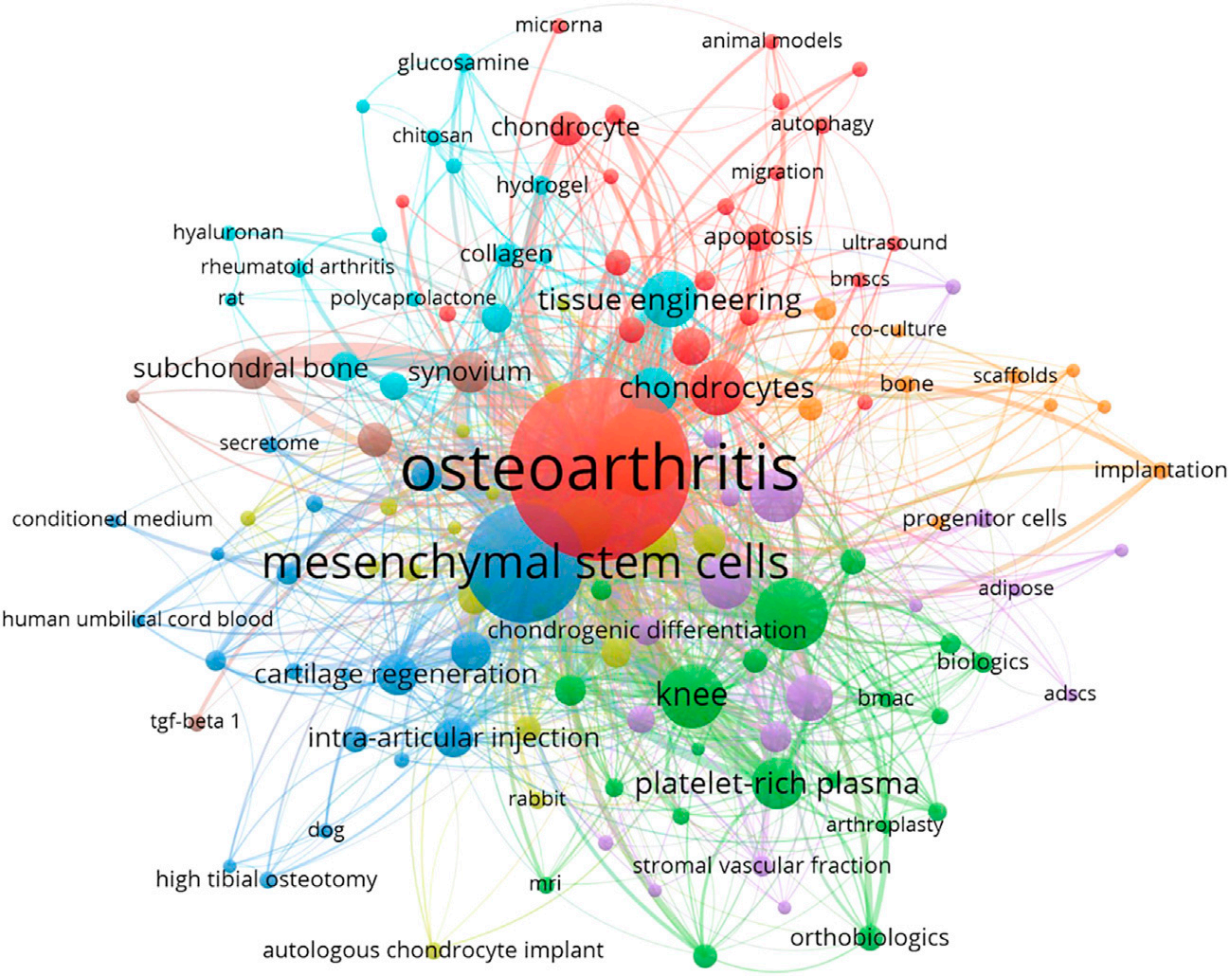
Co-occurrence analysis of all keywords regarding stem cell therapy for KOA research. Colors indicate different clusters.

Keywords, as a highly condensed version of article theme, is given co-occurrence analysis to detect the research direction or highlights. Of the 2215 keywords, 138 occurred more than five times. In the co-occurrence network visualization of the keywords (Figure 6), the top five keywords by occurrence weight were “osteoarthritis” (occurrences, 543; total link strength, 1,177), “mesenchymal stem cells” (occurrences, 243; total link strength, 616), “cartilage” (occurrences, 142; total link strength, 379), “stem cells” (occurrences, 97; total link strength, 287), and “knee” (occurrences, 79; total link strength, 245), in descending order (Table 3).

**TABLE 3:** Top 10 keywords by frequency from the included publications on stem cell research for KOA (*n* = 100).

Rank	Keyword	Total link strength	Occurrences	Avg. pub. year
1	Osteoarthritis	1,177	543	2017.54
2	Mesenchymal stem cells	616	243	2016.81
3	Cartilage	379	142	2017.00
4	Stem cells	287	97	2017.67
5	Knee	245	79	2017.80
6	Tissue engineering	178	65	2015.20
7	Cartilage repair	140	59	2016.14
8	Chondrocytes	146	59	2017.57
9	Chondrogenesis	120	56	2015.50
10	Articular cartilage	132	55	2016.13

Avg. pub. year: Mean year across publications (to the nearest two decimal places).

### Bibliometric analyses of theme terms and topic trends

**FIGURE 7:**
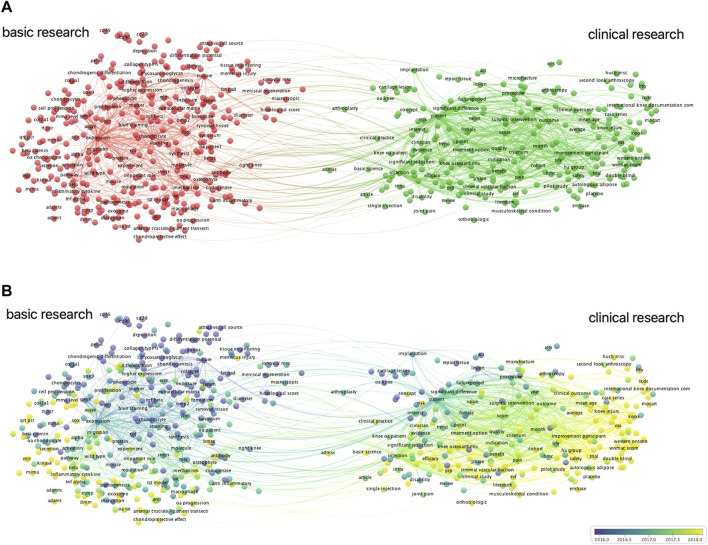
Co-occurrence analysis of all theme terms regarding stem cell therapy for KOA research. **(A)** Red cluster represents clinical research and green cluster represents basic research. **(B)** Co-occurrence of theme terms overlaying the visualization indicates the distribution of themes in different periods in clinical and basic research.

Among the 28,853 terms, 962 occurred more than 10 times. In the theme terms of the co-occurrence network visualization (Figure 7A), the red cluster represents the clinical study of stem cell therapy for KOA, and the green cluster stands for basic research. The co-occurrence overlay visualization theme terms indicate theme distribution across different periods in clinical and basic research (Figure 7B). The top 10 earliest and latest theme terms are listed in Table 4.

**TABLE 4:** Top 10 earliest and latest keywords of included publications on stem cell research for KOA (*n* = 100).

Earliest				Latest			
Rank	Theme terms	Occurrences	Avg. pub. year	Rank	Theme terms	Occurrences	Avg. pub. year
1	Fibroblast growth factor	16	2011.81	1	Extracellular vesicle	19	2020.37
2	Multipotentiality	10	2012.30	2	Exosome	25	2019.84
3	Osteophyte	12	2013.42	3	smscs	11	2019.27
4	Microarray analysis	13	2013.61	4	smsc	10	2019.10
5	Pellet culture	21	2013.90	5	col2	11	2019.09
6	Articular surface	17	2013.94	6	sem	11	2019.09
7	Pellet	33	2014.09	7	tnf	13	2019.07
8	Disruption	14	2014.14	8	Immunofluorescence	14	2019.00
9	Osteophyte formation	21	2014.19	9	oa development	14	2019.00
10	Hypertrophic chondrocyte	10	2014.30	10	mir	39	2018.97

Avg. pub. year: Mean year across publications (to the nearest two decimal places).

### Bibliometric analysis of keywords’ burst value

**FIGURE 8:**
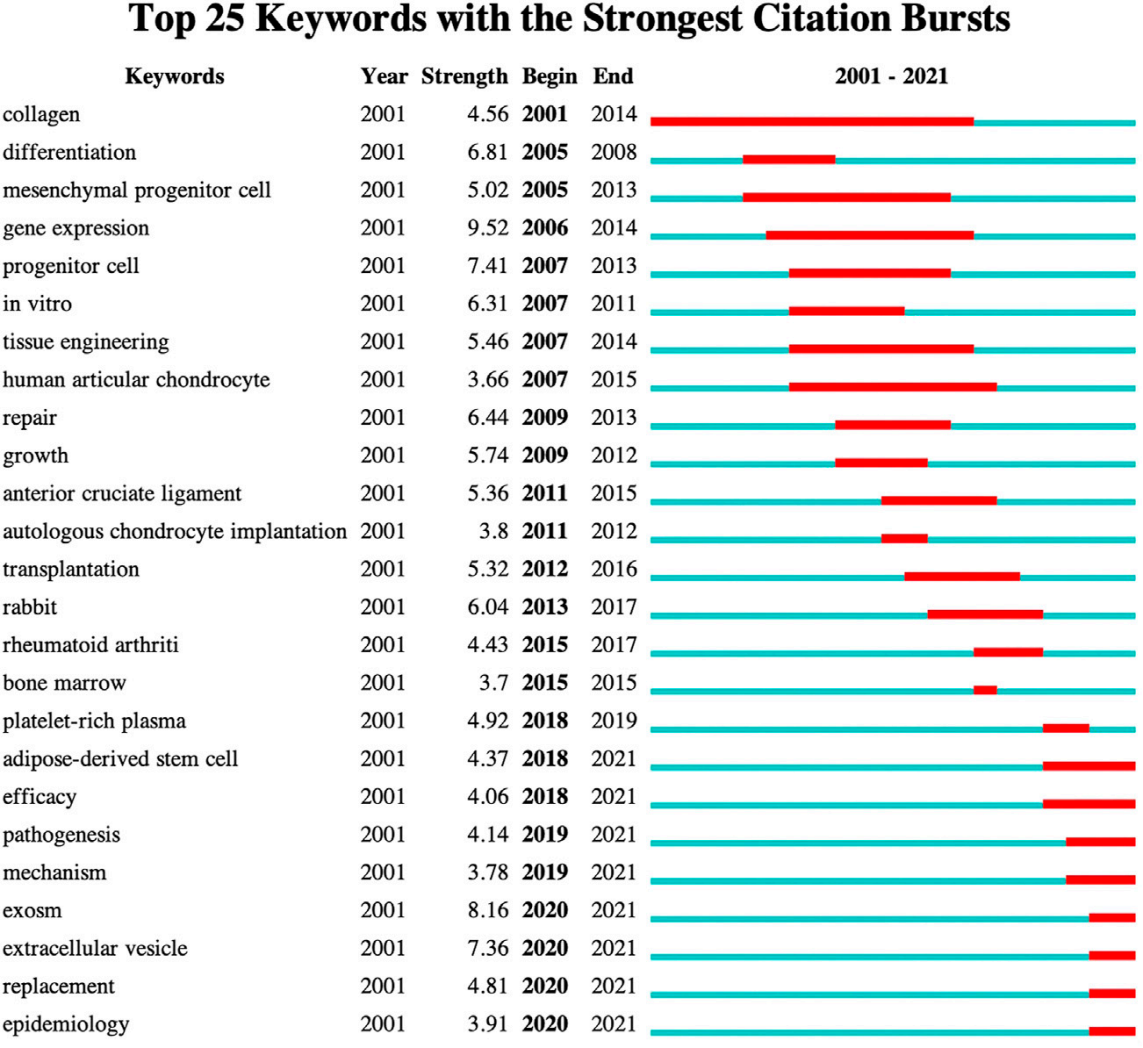
Top 25 keywords with the strongest burst value on stem cell therapy for KOA research. The dark blue bar shows years in which keywords received slight increases in co-occurrence, and the red bar indicates that co-occurrence rose sharply.

The keyword burst detection algorithm can identify keywords that have recently increased in popularity in the academic community. This approach displays the results in two dimensions: burst value and burst time. Keywords with high burst value in a given time period demonstrate that they have received special attention in the corresponding time interval and, to some extent, refer to the application frontiers of the research domain (Turatto et al., 2021). The top 25 keywords in the burst value are shown in Figure 8. From 2001 to 2017, gene expression, progenitor cell, differentiation, repair, and *in vitro* were commonly cited, with burst strengths reaching 9.52, 7.41, 6.81, 6.44, and 6.31, respectively. From 2015 to 2021, exosome, extracellular vesicle, platelet-rich plasma, replacement, and adipose-derived stem cell were commonly cited, with the burst strength reaching 8.16, 7.36, 4.92, 4.81, and 4.37 respectively.

### Bibliometric analysis of co-cited reference clustering, time evolution and burst value

**FIGURE 9:**
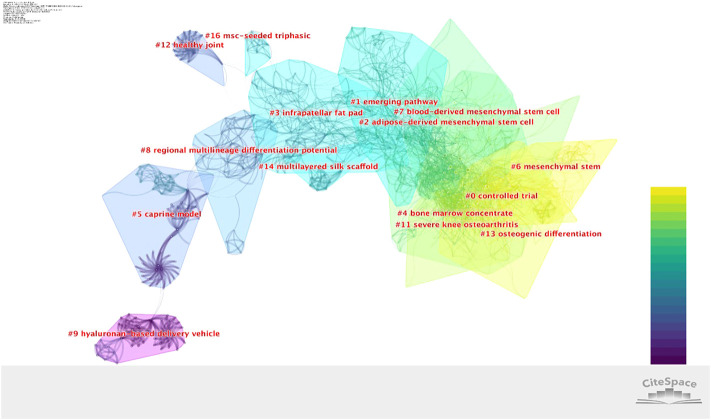
Analysis of co-cited reference clustering on stem cell therapy for KOA research. The color indicates the publication year. Vertical descending order indicates cluster size. The thickness of the line indicates the strength of the link.

**FIGURE 10:**
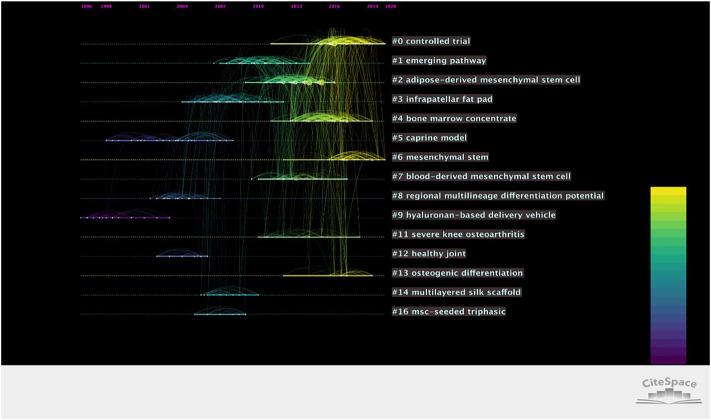
Time evolution analysis for co-cited references for stem cell therapy in KOA research. The color indicates the publication year. Colored curves represent co-citation links added for the year of the corresponding color. Large nodes indicate either large numbers of citations, presence of citation bursts, or both. Vertically descending order indicates cluster size. The publication year is indicated above the map.

As shown in Figures 9, 10 the 1,345 publications were divided into 21 clusters, for which the descending order vertically indicated cluster size. Cluster labels were obtained using the log likelihood ratio (LLR) and mutual information (MI). The modularity Q score was 0.83 higher than 0.4, meaning that the definition of every subdomain and the features of knowledge clusters differed greatly. The mean silhouette was 0.90 higher than 0.7, proving the efficiency and persuasiveness of the clustering effect. The top 10 most significant clusters were listed (Figure 9), with “controlled trial” being the most prominent. A timeline view was utilized to present the period of each cluster and the correlation between the different clusters, which demonstrated the evolution of stem cell therapy for KOA. In observations of the map, the “controlled trial” and “mesenchymal stem” were identified on the right side of the time axis, indicating their importance for research.

### Bibliometric analysis of co-cited reference’s burst value

**FIGURE 11:**
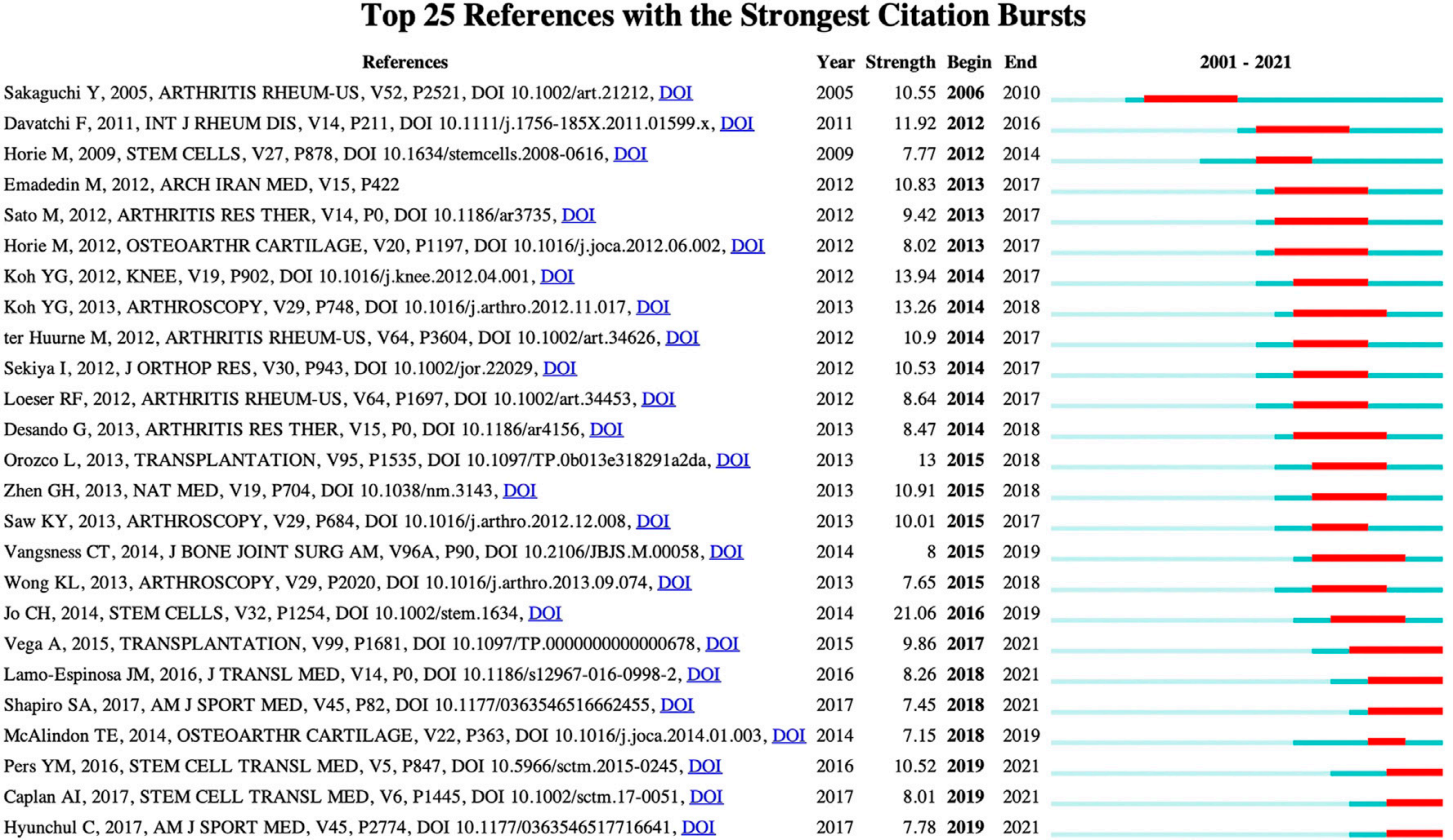
Top 25 cited references with the strongest burst value on stem cell therapy for KOA research. Dark blue bar indicates the years in which reference cited co-occurrence slightly increases. The red bar shows that reference cited rises sharply.

Citation bursts have been successfully applied to grasp the sharp increase in a relevant research direction. An upsurge in the frequency of a research citation over a time period is conceived as a mark of academic focus, which implies an underlying research trend (Chen et al., 2012). Therefore, citation burst detection was adopted to identify emerging trends. Figure 11 provides the top 25 references which show the strongest citation bursts. As shown from the chart, the dark blue bar represents the years in which citations slightly increased, while the red bar shows a sharp increase in citations. To probe deeper into these references, in this essay, only articles with the greatest link strength are discussed. Most top co-cited references are from specialty journals of *Stem Cells,* and these references have burst since 2016. From then, the publication “CH Jo, 2014, *Stem Cells*” (Jo et al., 2014) (strength: 21.06) had the highest burst strength, followed by “YG Koh, 2012, *Knee*” (Koh and Choi, 2012) (strength: 13.94), “YG Koh, 2013, *Arthroscopy*” (Koh et al., 2013) (strength: 13.26), “F Davatchi, 2011, *It J Rheum Dis*” (Davatchi et al., 2012) (strength: 11.92), and “GH Zhen, 2013, *Nat Med*” (Zhen et al., 2013) (strength: 10.91). The average burst has a duration of 2–4 years.

## Discussion

### Top-cited contributors of stem cells therapy for KOA

An epidemiological survey published in Proceedings of the National Academy of Sciences (PNAS) showed that the incidence of KOA in the U.S. had doubled since the mid-twentieth century (Wallace et al., 2017). Epidemiological research estimates that the lifetime risk of developing symptomatic KOA has reached 45% (Murphy et al., 2008). The shifting demographics consisting of an increasing number of American citizens who are above 65 years old, determines that the burden of KOA will keep growing (Prevention, 2013), and clinicians are urged to incorporate new treatment modalities, such as stem cell therapy. Citation analysis is extensively used to evaluate the quality of research work, as citation counts generally represent scientific acknowledgment by professionals (Zhong et al., 2021). This novel and promising treatment has received growing attention in recent decades as publications only appeared in one-digit numbers per year for the first 6 years since 2001, reaching two digits in 2007, and attaining over 150 publications in 2018. The development track of the past two decades can be separated into two stages: The 2001–2013 stage exhibited slow development, while the 2014–2021 stage picked up rapid development. Therefore, it is likely that more in-depth studies of the function of stem cells in treating KOA will be published in the coming years. The present optimistic results can in turn enable researchers to conduct studies with higher quality. Of the ten most productive individual countries and institutions, five were in Europe (Italy, Germany, the UK, Netherlands, Spain), three in Asia (China, Japan, South Korea), and two in North America (United States, Canada). Because stem cell therapy remains an immature technique, it is unsurprising that developed countries dominate the abovementioned list. This research also showed that the most productive country and institution were United States and Tokyo Med & Dent Univ separately. Thus, the United States and Japan are leaders in the field. *Osteoarthritis and Cartilage*, *American Journal of Sports Medicine*, *Journal of Orthopaedic Research*, and *Cartilage* published the largest number of studies on stem cells in treating KOA. As these journals were most favored by researchers around the world, their high reputations and authority regarding orthopedics research are implied. *Osteoarthritis and Cartilage* had the most publications and citations for cartilage repair, the most promising application of stem cells.

### International collaboration

The number of international collaborative articles was calculated. This cluster visualization may encourage wider collaboration among countries, either to reinforce the productivity of existing cooperation, or to stimulate new cooperation. These countries were also the most productive in terms of publications relating to OA. In stem cell therapy for KOA, China, the United States, the UK, and certain other Western countries and regions have shown close cooperation, but the lack of international cooperation in Asia has urged researchers to further strengthen research development in this field.

### Research hotspots and trends

The co-occurrence network visualization of theme terms has indicated that basic and clinical research complement each other, and basic research accelerates the transformation of stem cell therapy into clinical application. Among the top 10 latest theme terms, as shown in Table 4, MSCs and exosome vesicles (including exosomes) were identified as attracting more attention in current research. By refining the research topics of articles through a keyword research, it was found that during the last 20 years, the primary research direction of stem cell therapy for the KOA field focused on the mechanism of cartilage repair (Figure 8). As is known, OA gets mediated by a complex but not yet fully researched interplay of proinflammatory and anti-inflammatory cytokines, chemokines, growth factors. and adipokines that can all be measured in the serum, potentially functioning as biomarkers of disease stage and progression, synovium, and histological samples. The epigenome that regulates all the genetic expression through DNA methylation, histone modifications, and mRNA interference is deemed as another key aspect of disease progression (Primorac et al., 2020). The mechanism of stem cell therapy includes the unique ability of self-renewal and multi-directional differentiation, which enables cells’ infinite proliferation and differentiation into cartilage under specific conditions. In addition, MSCs can release a variety of growth factors and cytokines, deliver paracrine, and provide anti-inflammatory and immunomodulatory effects. It can establish a desirable micro-environment for tissue repair as well (Caplan, 2019). Through the analysis of research hotspots and citation burst, gene expression, differentiation, repair, and exosome and extracellular vesicles were identified as research hotspots that coincide with the therapeutic mechanism of stem cells. Based on co-cited reference clustering analysis (Figure 10), the research trend of stem cells for KOA was correlated with the terms “controlled trial” and “mesenchymal stem.” “Osteogenic differentiation” remains the focus of recent basic research. It is speculated that mesenchymal stem cells are the main players in cell therapies, including those on exosomes from which they are derived. While “emerging pathway” ranks second on the co-cited reference cluster list, it has not yet become a research highlight. Analysis of research hotspots and trends, it is not difficult to find that the research direction of stem cell therapy for KOA seems to be carried out with the continuous discovery of the pathogenesis of OA.

### Strengths and limitations

This research serves as the first bibliometric analysis to evaluate publications on the application of stem cell therapy for the KOA field extracted from the WoS Core Database, maintaining objectivity and comprehensiveness in the data analysis. This research provides a large quantity of information illustrating the status quo, hotspot issues, and outlook for stem cell therapy in KOA research as well. Additionally, to clearly demonstrate the bibliometric results, a variety of visualization tools were employed. Nevertheless, inevitable limitations occurred. First, the bibliometric analysis failed to analyze different languages as a whole. Second, only original articles were included. Third, some words were counted several times owing to their appearance in multiple expressions. Finally, all the database searches were finished in one single day to attempt avoiding bias generated by updated publications. However, new data may have gone missing as well, although its impact on citation frequency can be ignored.

## Conclusion

In conclusion, the United States leads the research field. Further analysis shows that research mainly focuses on mechanisms of cartilage repair. Furthermore, to detect more MSCs involved in cartilage regeneration that have not yet been identified, further relevant studies are essential to verifying MSCs’ role as a viable KOA treatment option. Further studies of stem cells in KOA, particularly those involving exosomes for KOA, are expected to attract continued attention. For clinical translation, as MSCs are the most widely explored stem cell types, more experimental and clinical studies ought to facilitate the clinical application of stem cells.

## Data Availability

The original contributions presented in the study are included in the article/Supplementary Material; further inquiries can be directed to the corresponding authors.
